# Single- versus two- layer intestinal anastomosis: a meta-analysis of randomized controlled trials

**DOI:** 10.1186/1471-2482-6-2

**Published:** 2006-01-27

**Authors:** Satoru Shikata, Hisakazu Yamagishi, Yoshinori Taji, Toshihiko Shimada, Yoshinori Noguchi

**Affiliations:** 1 Department of Digestive Surgery, Kyoto Prefectural University of Medicine, Kyoto, Japan; 2 Department of Clinical Epidemiology, Kyoto University, Kyoto, Japan; 3 Department of Medicine, Fujita Health University School of Medicine, Aichi, Japan

## Abstract

**Background:**

To compare single- with two- layer intestinal anastomosis after intestinal resection: a meta-analysis of randomized controlled trials.

**Methods:**

Randomized controlled trials comparing single- with two-layer intestinal anastomosis were identified using a systematic search of Medline, Embase and the Cochrane Library Databases covering articles published from 1966 to 2004. Outcome of primary interest was postoperative leak. A risk ratio for trial outcomes and weighted pooled estimates for data were calculated. A fixed-effect model weighted using Mantel-Haenszel methods and a random-effect model using DerSimonian-Laird methods were employed.

**Results:**

Six trials were analyzed, comprising 670 participants (single-layer group, n = 299; two-layer group, n = 371). Data on leaks were available from all included studies. Combined risk ratio using DerSimonian-Laird methods was 0.91 (95% CI = 0.49 to 1.69), and indicated no significant difference. Inter-study heterogeneity was significant (χ^2 ^= 10.5, d.f. = 5, p = 0.06).

**Conclusion:**

No evidence was found that two-layer intestinal anastomosis leads to fewer post-operative leaks than single layer. Considering duration of the anastomosis procedure and medical expenses, single-layer intestinal anastomosis appears to represent the optimal choice for most surgical situations.

## Background

The basic principles of intestinal suture were established more than 100 years ago by Travers, Lembert and Halsted [1], and have since undergone little modification. Development of stapling instruments for intestinal anastomosis has added new dimensions to intestinal surgery. Two systematic reviews of randomized controlled trials (RCTs) comparing stapled with hand-sewn colorectal anastomosis found no difference between the two methods [2,3], but colorectal surgeons need to be familiar with both. Unsurprisingly, hand-suturing techniques were shown to display a longer learning curve than stapling [4]. One aspect of intestinal suturing technique that has remained controversial is the use of either one or two layers of sutures for anastomosis.

Historically, two-layer anastomosis using interrupted silk sutures for an outer inverted seromuscular layer and a running absorbable suture for a transmural inner layer has been standard for most surgical situations. Some recent reports have described single-layer continuous anastomosis using monofilament sutures as requiring less time and cost than any other method, without incurring any added risk of leakage [5-8]. Many surgeons probably now use single-layer suturing due to reductions in ischemia, tissue necrosis or narrowing of the lumen compared to the two-layer method.

While numerous RCTs have addressed this issue, no published reports have described meta-analysis of RCTs to date [9-14]. We therefore performed a meta-analysis of RCTs to assess efficacy and safety for single- and two-layer anastomosis after intestinal resection.

## Methods

### Search strategy and selection criteria

A search of any language literature was performed through August 2004 to identify reports of randomized controlled trials comparing single- with two-layer intestinal anastomosis. Data bases searched were Medline (1966-April 2004), the Cochrane Register of Controlled Trials (Issue2, 2004), and EMBASE(1986-August 2004). A detailed description of the search strategy is provided in the Appendix [see Additional file 1]. Electronic searches were supplemented by hand searching reference lists and reviews.

This meta-analysis included studies clearly describing the following: 1) study design (randomized controlled trial); 2) main outcome (effectiveness of single-layer vs. two-layer intestinal anastomosis); 3) target population (patients needing intestinal resection); and 4) availability of leak data. Studies that were not RCTs were excluded from the analysis.

### Data abstraction and quality assessment

Each investigator decided independently which reports should be included for analysis. Any disagreement was settled by consensus between all investigators. Data were extracted independently by two investigators (SS and YT), with any disagreements resolved by a final reviewer (YN).

Outcome of primary interest was risk of leakage related to intestinal anastomosis. Secondary outcomes comprised mortality, duration of anastomosis procedure, duration of total parenteral nutrition (TPN), length of hospital stay, risk of wound infection, and cost of sutures.

Quality of primary studies was evaluated as described by Jadad et al [15]. This method assesses description of randomization, appropriateness of randomization, description of double-blinding, appropriateness of double-blinding, and description of withdrawals and dropouts with scores of 0 or 1 for each. Minimum possible score was 0 and maximum was 5.

### Statistical analysis

The fixed-effect model weighted using Mantel-Haenszel methods was used for pooling the risk ratio [16], followed by a test of homogeneity. Inter-study homogeneity was assessed using the χ^2 ^test (Q statistics)[17]. A homogeneity value of P < 0.10 was considered statistically significant. If the hypothesis of homogeneity was rejected, the random-effect model using DerSimonian-Laird methods was employed [18].

Meta-regression analyses were performed to explore sources of heterogeneity. Variables comprising year of publication, mean age of study participants, and percentage of male patients were examined for significant effects on risk of leak. In addition to that, informal graphical exploration using a L'Abbe plot was made and sensitivity analysis was performed [19,20]. And another sensitivity analysis was performed by excluding low quality studies (studies with 1 point on the Jadad scale) to assess the impact of study quality. We then performed the other analysis by including studies in which operations were only performed by consulting surgeons, staff surgeons, or residents with 5 years or more experience.

The potential for publication bias was examined using the funnel plot method [21], and the significance of differences was evaluated in accordance with the methods described by Begg and Egger [22,23]. A value of P < 0.10 for publication bias was considered statistically significant. Risk ratios were calculated for trial outcomes and weighted pooled estimates for data.

All statistical analyses were performed using STATA statistical software version 8.1 (Stata Corporation, College Station, TX, USA). We used a user-written add-on Stata routine "metan", which was written by Bradburn et al.[24]. Results are expressed as mean with 95% confidence intervals (CIs). Values of P < 0.05 were considered statistically significant unless otherwise indicated.

## Results

### Study characteristics

**Figure 1:**
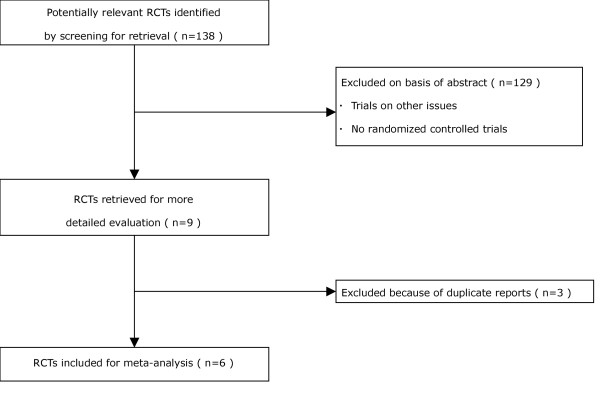
**Flow of retrieval and inclusion of randomised controlled trials for meta-analysis**. RCT = randomized controlled trial.

Figure 1 shows a summary profile of the search. A database search yielded 138 articles, and manual search of bibliographies in these articles yielded no additions. Of the 138 articles, 9 met all inclusion criteria. A further 3 studies that were considered to represent multiple publications were excluded. A total of 6 studies were therefore analyzed. Agreement between authors for selection of relevant articles was unanimous.

The 6 trials included for analysis comprised 670 participants (single-layer group, n = 299; two-layer group, n = 371). Table 1 shows baseline characteristics for participants in the included studies. Mean age in the studies by Ordorica et al. and Maurya et al., 4 years and 30 years, respectively, were rather young compared to the remaining studies. The size of the two-layer group was double that of the single-layer group in the study by Maurya et al. All primary studies met the inclusion criteria, but examined studies displayed differences in inclusion and exclusion criteria, definitions of terms and suture techniques (Tables 2, 3, 4).

Mean Jadad score was 1.7 (range, 1–3) (Table 1). None of the studies except that by Ordorica et al. met the requirements for appropriateness of double-blinding.

### Leaks

**Figure 2:**
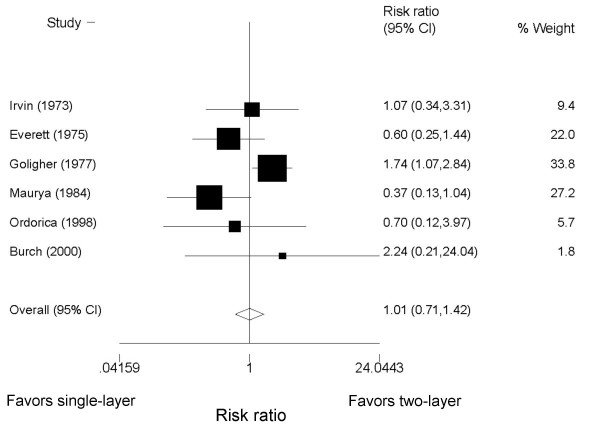
Risk ratios with 95% confidence intervals for postoperative leaks in prospective randomized controlled trials addressing single- vs. two-layer intestinal anastomosis.

Data on leaks were available from all included studies. In the study by Goligher et al., almost half of the cases that had been allocated to the single-layer group experienced leakage (31 of 69 patients). Included studies showed a tendency toward total risk of leaks decreasing annually (Table 1). Combined risk ratio using DerSimonian-Laird methods was 0.91 (95% CI = 0.49 to 1.69), indicating no statistical significance (Fig. 2). Inter-study heterogeneity was considered statistically significant (χ^2 ^= 10.5, d.f. = 5, p = 0.06).

### Other outcomes

For other outcomes, no meta-analysis could be performed due to lack of sufficient data. Data on mortality related to intestinal anastomosis, reported only in the study by Irvin et al, was equal in both groups. The arithmetical mean duration of anastomosis procedure in two included studies was 23.4 min vs. 36.9 min (single vs. two-layer), and arithmetical mean length of hospital stay was 9.9 days vs. 13.0 days. Data on duration of TPN, reported only in the study by Maurya et al., showed that the single-layer group could tolerate oral fluids earlier, and duration of intravenous alimentation was shorter compared to two-layer patients (4.8 days vs. 6.7 days, respectively). No study reported risk of wound infection. Suture cost was reported only in the study by Burch et al., and was $4.5 for single-layer compared to $35.4 for two-layer anastomosis.

### Exploring sources of heterogeneity and sensitivity analysis

**Figure 3:**
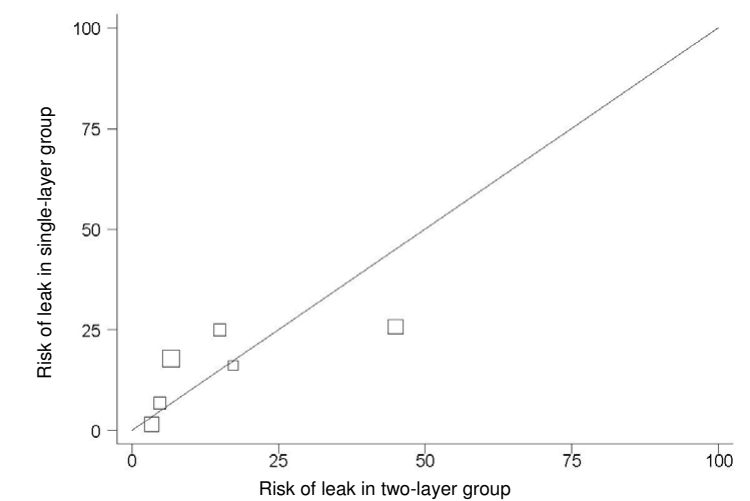
L'Abbe plot of risk of leak in single-layer vs. two-layer.

Meta-regression revealed that neither year of publication, mean age of study participants, nor percentage of male patients were related to risk of leak. From the graphical exploration using a L'Abbe plot [Fig. 3], we considered the study by Goligher et al. as the source of heterogeneity and we performed sensitivity analysis excluding this study. With the fixed-effect model, the combined risk ratio for leak was 0.63 (95%CI = 0.37 to 1.06) and inter-study heterogeneity was not significant (χ^2 ^= 2.96, d.f. = 4, p = 0.57). Another sensitivity analysis was performed including only high-quality studies (n = 3) with a Jadad score ≥2. The combined risk ratio for leak was 1.65 (95% CI = 1.04 to 2.61) with DerSimonian-Laird estimate, indicating more favorable results for two-layer anastomosis compared to single. In two included studies by Everett et al. and Ordorica et al., operations were performed only by consulting surgeons, staff surgeons, and residents of ≥5 years experience. Combined risk ratio for leak was 0.61 (95% CI = 0.28 to 1.35), and no significant difference was found.

### Publication bias

Funnel-plot, Begg's test and Egger's test were performed to evaluate potential for publication bias in terms of leakage rate related to intestinal anastomosis. The funnel-plot showed a symmetrical pattern, and neither statistical test revealed the presence of significant publication bias (Begg's test, P = 1.00, Egger's test, P = 0.50).

## Discussion

The present study assessed the efficacy and safety of single- and two-layer anastomosis after intestinal resection. The main finding of the study was that there is no evidence of a difference in terms of risk of leak but that there is insufficient evidence to rule out a modest but potentially important difference. Sensitivity analysis excluding the study by Goligher et al. suggested it as the source of heterogeneity. In their trial, techniques of vertical mattress sutures in the posterior two-thirds of the circumferences and Lembert sutures of horizontal mattress type in the anterior third of the bowel circumference were performed in single-layer group and reported the highest risk of leaks (45%). One possible explanation of this high rate of leaks may be their inclusion criteria, high and low colorectal anastomosis. On this subject, they described "We are quite unable to explain the difference between Everett's results and ours" in their report [11]. This suture technique is not common in intestinal anastomosis in the present day. Although various endpoints can be used to assess efficacy and safety of intestinal anastomosis, risk of leak after operation occupies the greatest attention among surgeons. Because there is no difference in the main outcome between two techniques, choices in clinical practice should be made after taking into account the results of other outcomes such as mortality, duration of anastomosis procedure, duration of TPN, length of hospital stay, risk of wound infection, and cost of sutures. Arithmetical means of these endpoints suggests that the single-layer method offers almost the same or better results than the two-layer method.

None of the studies except Ordorica et al. met the requirements for appropriateness of double-blinding. In the study by Ordorica et al., neither the physician performing the assessments nor the pediatric patient knew the type of anastomosis. However, assessing outcomes under blinding is virtually impossible in surgical trials. We therefore regarded studies with a Jadad score of 3 as high-quality studies.

### Limitations

There are several limitations in current study. First, the study by Goligher et al. had a substantial influence on the combined risk ratio. However, the main conclusion of a lack of evidence for an advantage of two-layer over single-layer anastomosis is unaffected, as the result of a sensitivity analysis excluding this study was more favourable to single-layer. Secondly, the quality of individual RCTs included in our analysis was not necessarily high, mainly due to a lack of blinding. This is, however, inevitable in most surgical trials. Thirdly, there were differences in inclusion criteria, definition of the term "leak" and suture techniques for studies included for meta-analysis. Exclusion criteria among studies also varied. Lastly, the total number of patients included for this meta-analysis might not have been sufficient to identify small differences between the two techniques. However, no significant differences in methods were identified. Future RCTs may yield different conclusions from meta-analysis. Despite these limitations, we believe that this meta-analytic overview provides the current best information in making clinical decision to choose a surgical suture techniques.

## Conclusion

The current meta-analysis clarified that two-layer intestinal anastomosis offers no definite advantage over single-layer anastomosis in terms of postoperative leak. Considering duration of the anastomosis procedure and medical expenses, single-layer intestinal anastomosis may prove the optimal choice in most surgical situations.

## Abbreviations

RCTs = randomized controlled trials

TPN = total parenteral nutrition

CI = confidence interval

## Competing interests

The author(s) declare that they have no competing interests.

## Authors' contributions

SS conceived the study, participated in the design of the study, searched the reports, selected and evaluated reports, performed data abstraction and drafted the manuscript. HY and TS participated in the design of the study, searched the reports, selected and evaluated reports. YT participated in the design of the study and performed the data abstraction and statistical analysis. YN participated in its design and coordination and helped to draft the manuscript. All authors read and approved the final manuscript.

## Pre-publication history

The pre-publication history for this paper can be accessed here:



## Supplementary Material

Additional File 1 A detailed description of the search strategy is provided in this file. Click here for file

## Figures and Tables

**Table 1:** Characteristics and outcomes of the studies included for meta-analysis.

				Single-/Two-layer							
								
								No. of leaks/patient (%)		No. of death/patient (%)	
									
Authors (ref.)	Year	Country	Jadad score	Mean age (yr)	Males (%)	Mean duration of anastomosis (min)	Mean length of hospital stay (day)	Single-	Two-layer	Single-	Two-layer
Irvin et al. (9)	1973	U.K	1	64.0/57.0	41.4/54.8	NR	NR	5/29 (17)	5/31 (16)	3/29 (10)	3/31 (10)
Everett et al. (10)	1975	U.K	1	64.0/62.5	43.1/47.0	NR	NR	6/40 (15)	13/52 (25)	NR	NR
Goligher et al. (11)	1977	U.K	2	63.4/63.5	42.0/37.9	NR	NR	31/69 (45)	17/66 (26)	NR	NR
Maurya et al. (12)	1988	India	1	29.8/31.6	60.0/62.5	NR	11.4/18.6	4/60 (7)	20/112 (18)	NR	NR
Ordorica et al. (13)	1998	Mexico	3	3.7/3.7	NR	26/43	10.4/10.4	2/42 (5)	3/44 (7)	NR	NR
Burch et al. (14)	2000	U.S.A	2	44.3/44.7	64.6/59.7	20.8/30.7	7.9/9.9	2/59 (3)	1/66 (2)	NR	NR

NR = not reported

**Table 2:** Inclusion and exclusion criteria in the studies included for meta-analysis.

Author (ref.)	Year	Inclusion criteria	Exclusion criteria
Irvin (9)	1973	Resection with end-to-end anastomosis of the small or large intestine	NR
Everett (10)	1975	Elective resection of the large bowel and end-to-end colorectal anastomosis	colo-anal anastomosis, extra-peritoneal anastomosis
Goligher (11)	1977	High and low colorectal anastomosis following resection for carcinoma	NR
Maurya (12)	1984	With bowel resection and end to end anastomosis only	NR
Ordorica (13)	1998	Pediatric, aged between 1 month and 16 years	duodenum, rectum, enteroplasty or proximal stoma
Burch (14)	2000	Requiring intestinal anastomosis	duodenum, rectum/surgeon's technical concerns

NR = not reported

**Table 3:** Definition of leak and operator employed in the studies included for meta-analysis.

Author (ref.)	Year	Leak	Operator
Irvin (9)	1973	Barium enema examination was performed on the 14th postoperative day	NR
Everett (10)	1975	Barium enema examination was performed on the 10th postoperative day	Only by the author, consultant surgeon
Goligher (11)	1977	Gastrografin enema study was carried out to secure a visual record of leak	NR
Maurya (12)	1984	NR	NR
Ordorica (13)	1998	NR	Staff surgeons and residents of 5th and 6th grade
Burch (14)	2000	Fistula radiographically or a visible disruption of the suture line during reexploration	Residents of 3rd to 5th grade or attending surgeon

NR = not reported

**Table 4:** Suture techniques of the studies included for meta-analysis.

	Single-layer		Two-layer			
			
			inner		outer	
				
Author (ref.)	technique	material	technique	material	technique	material
Irvin (9)	12:submucosal, 6:all-layer, 11:Gambee	silk	continuous all layer	chromic catgut	interrupted Lembert	silk
Everett (10)	Gambee	4-0 Supramid	continuous all layer	2-0 chromic catgut	interrupted Lembert	4-0 Supramid
Goligher (11)	vertical mattress and Lembert	3-0 silk	continuous all layer	3-0 chromic catgut	interrupted Lembert	3-0 silk
Maurya (12)	Gambee	silk	continuous all layer	3-0 chromic catgut	interrupted Lembert	3-0 silk
Ordorica (13)	Gambee	4-0 or 5-0 poliglactine	connel-Mayo	4-0 or 5-0 poliglactine	interrupted Lembert	4-0 or 5-0 poliglactine
Burch (14)	all layer-continous	3-0 polypropylene	continuous transmural	3-0 polyglycoic acid	interrupted Lembert	3-0 silk
