# Supplementary material for: Single- versus two- layer intestinal anastomosis: a meta-analysis of randomized controlled trials
Source: BMC Surg. 2006 Jan 27;6:2. doi: 10.1186/1471-2482-6-2 (PMC1373646; doi:10.1186/1471-2482-6-2)
Supplement: Additional File 1 — A detailed description of the search strategy is provided in this file. [file 1471-2482-6-2-S1.doc]

Additional file 1: Appendix - Search strategy.doc

**Single- Versus Two- Layer Intestinal Anastomosis:**

**A Meta-Analysis of Randomized Controlled Trials**

**Appendix**

**Search strategy**

A review of the literature was performed in April 2004 to produce a bibliography of articles, abstracts, and research reports in which comparing single- with two-layer intestinal anastomosis was studied in a randomized controlled trial. The following databases were searched: MEDLINE (PubMed) and the Cochrane Central Register of Controlled Trials. Additionally, searches were performed in EMBASE August 2004. Studies were retrieved regardless of language. Electronic searches were supplemented by hand searching reference lists and reviews.

MEDLINE is the premier biomedical bibliographic database, produced by the National Library of Medicine (NLM). NLM indexes over 4600 worldwide life science journals. MEDLINE includes over 12 million references from 1966 to the present. Indexers assign Medical Subject Headings (MeSHs), NLM’s controlled thesaurus of descriptors. Most of the references starting in 1975 also contain an abstract. MEDLINE was searched using the PubMed search interface.

The following search strategy was used:

#1 research design [mh] OR clinical trials [mh] OR comparative study [mh] OR placebos [mh] OR multicenter study [pt] OR clinical trial [pt] OR random* [tiab] OR placebo*[tiab] OR clinical trial* [tiab] OR controlled clinical trial [pt] OR randomized controlled trial [pt] OR practice guideline [pt] OR feasibility studies [mh] OR clinical protocols [mh] OR single blind* [tiab] OR double blind* [tiab] OR triple blind* [tiab] OR treatment outcomes [mh] OR epidemiologic research design [mh] OR double blind method [mh] OR pilot projects [mh]

#2 "Anastomosis, Surgical"[MeSH]

#3 "Intestines"[MeSH] OR"colon" OR"small intestine"

#4 "one layer"OR "one-layer"OR "a layer"OR"single layer"OR"single-layer" OR"two-layer"

#5 #1AND((#2 AND #3) OR #4)

The Cochrane Central Register of Controlled Trials (CCTR) is a component of the Cochrane Evidence-Based Medicine Reviews Collection. It is a bibliographic database of definitive controlled trials identified by the contributors to the Cochrane Collaboration. Cochrane groups and other organizations contribute their specialized registers, and together with references to clinical trials identified in MEDLINE and EMBASE, form the CCTR database. CCTR was searched using the following search strategy:

#1 [SUTURE TECHNIQUES explode tree 1 (MeSH)](http://212.49.218.200/newgenMB/ASP/srchResults.asp?histNo=1)

#2 [INTESTINES explode tree 1 (MeSH)](http://212.49.218.200/newgenMB/ASP/srchResults.asp?histNo=2)

#3 [(#1 and #2)](http://212.49.218.200/newgenMB/ASP/srchResults.asp?histNo=3)

The Excerpta Medica Database (EMBASE) is a biomedical and pharmacological resource providing access to the most up-to-date information about medical and drug-related subjects. It covers the literature from 1974 to the present. EMBASE provides access to periodical articles from more than 3,700 primary journals from approximately 70 countries. EMBASE was searched using the following search strategy:

1. intestin? Or colo?
2. random? And trial? ?
3. 1 and 2

We also searched the reference lists from the primary studies and review articles, sought help from experts in the field.

The subsequent criteria for selection of trials to be included in the meta-analysis are described in the Methods section, and the flowchart of trials is presented in Figure 1.
